# One-Pot Graphene Supported Pt_3_Cu Nanoparticles—From Theory towards an Effective Molecular Oxygen Reduction Reaction Catalyst

**DOI:** 10.3390/molecules28135072

**Published:** 2023-06-28

**Authors:** Carlos Daniel Galindo-Uribe, Gerald Geudtner, Patrizia Calaminici, Omar Solorza-Feria

**Affiliations:** Chemistry Department, Research Center for Advanced Studies (CINVESTAV), Av. Instituto Politécnico Nacional 2508, Col. San Pedro Zacatenco, Del. Gustavo A. Madero, Mexico City 07360, Mexico; carlosd.galindo@cinvestav.mx (C.D.G.-U.); geudtner@cinvestav.mx (G.G.)

**Keywords:** nanoparticles, graphene, ORR, ADFT, growing pattern, electrocatalyst, Pt_3_Cu alloy

## Abstract

In this work, recent research progresses in the formation of Pt_3_Cu nanoparticles onto the surface of graphene are described, and the obtained results are contrasted with previously published theoretical studies. To form these nanoparticles, tetrabutylammonium hexachloroplatinate, and copper acetylacetonate are used as platinum and copper precursors, respectively. Oleylamine is used as a reductor and a solvent. The obtained catalyst is characterized via X-ray diffraction (XRD), transmission electron microscopy (TEM), scanning electron microscopy (SEM), and energy dispersive spectroscopy X-ray (EDS). To assess the catalytic activity, the graphene-supported Pt_3_Cu material is tested with cyclic voltammetry, “CO stripping”, and oxygen reduction reaction potentiodynamic curves to find the nature and the intrinsic electrochemical activity of the material. It can be observed that the tetrabutylammonium cation plays a critical role in anchoring and supporting nanoparticles over graphene, from which a broad discussion about the true nature of the anchoring mechanism was derived. The growth mechanism of the nanoparticles on the surface of graphene was observed, supporting the conducted theoretical models. With this study, a reliable, versatile, and efficient synthesis of nanocatalysts is presented, demonstrating the potentiality of Pt_3_Cu/graphene as an effective cathode catalyst. This study demonstrates the importance of reliable ab inito theoretical results as a useful source of information for the synthesis of the Pt_3_Cu alloy system.

## 1. Introduction

Humanity today faces two important crises: the climate change crisis and the energy crisis. To address these crises together is critical to replace the fossil fuel technologies existing today with more environmentally friendly technologies [1]. Among these technologies, the polymer exchange membrane fuel cell (PEMFC) is one of the most promising technologies available. The PEMFC has significant advantages such as a high energy density and low temperature [2,3,4,5,6,7,8], which makes it an ideal candidate for portable, stationary, residential, and transport applications.

However, the use of PEMFCs is hindered by their high cost and longtime instability. Most of the cost is because the PEMFC uses catalysts made from precious metals, such as Pt [9]. Therefore, the design of new novel materials is of the utmost importance to the massive adoptions of technologies that can help in the transition to renewable energies.

To diminish the content of Pt, which is an expensive and scarce element on our planet, as well as the overall cost of the PEMFC, it is important to improve the catalyst material design. Over the last years, different approaches have been used, such as controlling the geometry, morphology, size, and porosity of the used nanoparticle [10] and alloying Pt with different non-noble transition metals, such as Fe, Ni, Co, and Cu, among others [9]. It is also essential to ration the use of precious materials such as Pt, Pd, Au, etc., exchanging them for non-noble and more abundant metals. The studies for novel capacitors and electrolyzers show that a replacement of these materials is possible and successful [11,12]; therefore, this tendency should also be adopted into the PEMFC catalysts. Of all possible materials that alloys formed with Pt and the combinations of different transition metal elements, the Pt_3_Cu alloy has attracted a lot of attention in recent years as a versatile catalyst. Recent studies show that Pt_3_Cu can adopt different geometries as a nanocatalyst, such as an octahedron [13], twinned icosahedron [13], dendritic pyramid [14], wires [15], nanoframes [16], among others. The vast array of geometries obtained with this special alloy has been used extensively as catalysts for different reactions, such as the oxygen reduction reaction (ORR) [13,16,17], the methanol oxidation reaction [18], and the ethanol oxidation reaction [19].

**Figure 1:**
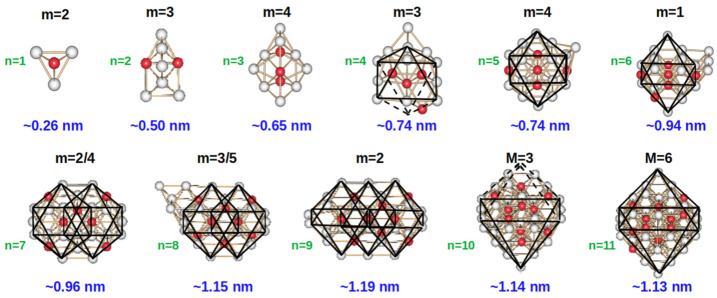
Most stable (Pt_3_Cu)_n_, n = 1–11 cluster structures along with their multiplicity and approximate size (in nm). The letter n indicates the number of Pt_3_Cu units with which each cluster size was initially formed. A black frame is added on the top of the cluster structures from n = 5 to n = 11 to guide the eye of the reader to observe the alternate formation of octahedra and twinned octahedra moieties.

To gain a better insight into the growth mechanism and energy properties of this system, we previously performed a series of very extended theoretical studies on the structures and properties of (Pt_3_Cu)_n_, n = 1–11 clusters, moving from a system size of a few atoms to the nanometer scale. These results and the employed methodology were published in Refs. [20,21]. These studies demonstrated that in the most stable (Pt_3_Cu)_n_, n = 5–6 cluster structures, the formation of structural single octahedra moieties was observed. However, in the (Pt_3_Cu)_n_, n = 7–9 cluster structures, the formation of twinned octahedra structures was preferred. Surprisingly, this tendency was broken at the (Pt_3_Cu)_n_, n = 10–11 cluster sizes, in which the obtained most stable geometries were also octahedra-based moieties. Moreover, the preference of Cu atoms to occupy central sites in the cluster structures and the formation of an inner smaller octahedron made by Cu atoms in the biggest (Pt_3_Cu)_11_ cluster structure was also observed, suggesting that core–shell structures for this type of systems are possible and stable [21]. Additionally, in our previous theoretical investigation, spin density plots were obtained, showing that the unpaired electrons in these systems are preferentially allocated to the Pt atoms [20,21]. The structures of the most stable clusters together with the growing pattern that we observed for these systems are graphically illustrated in Figure 1.

Figure 1 shows the most stable geometries obtained for the (Pt_3_Cu)_n_, n = 1–11 clusters, along with their electronic spin multiplicity and their approximate size in nm. The number of Pt_3_Cu units with which each cluster was initially formed, previous to its non-constrained full geometry optimization, is indicated with the letter n. It can be observed that the preferred spin multiplicity is 3 for the clusters formed with an even number of Pt_3_Cu units, whereas the preferred spin multiplicity is 2 and 4 for the clusters formed with an odd number of units. It is also important to note that this study of clusters included systems that are larger than 1 nm (the largest cluster size we studied with the employed ab-initio methodology was approximately 1.15 nm), which is a critical size for obtaining the properties of the nanoparticles (NPs) of the same alloy.

However, still no information about the bonding and connectivity of the atoms of these clusters is known, which is important to confirm the formation of a stable alloy bond. Therefore, one of the main objectives of this study is to first analyze the results of the molecular electrostatic potential (MEP) of the minimum energy structures of the (Pt_3_Cu)_n_, n = 1–11 clusters. From the analysis of the critical points (CP) of the MEP, a bond path can be calculated [22,23,24]. It is important also to mention here that these kinds of scalar fields, e.g., MEP and electron density, are measurable by experimental methods, such as X-ray and neutron diffraction [25,26]. Therefore, these studies could serve as a reference for future experiments that could be performed on this alloy. The analysis of MEP CPs has been extensively used to find the bond order and connectivity of numerous systems, such as metal and non-metal clusters [27,28], transition metal complexes [29], and alkali metal clusters [30,31], as documented in the literature.

Moreover, to experimentally obtain the best possible catalyst, is important to finely tune the NP size to improve the electrochemical surface area (ECSA). In this sense, the other objective of the study is to experimentally synthesize the nanoparticles with the structures proposed in the theoretical studies as the most stable geometries for the Pt_3_Cu alloy, i.e., octahedra and twinned octahedra [20,21], and convert them into a fully functional ORR catalyst. To achieve this objective, a support material is required to optimize the precious metal NPs content. In this context it is important to mention that this is a critical aspect concerning the durability of the catalyst [32]. Most of the support materials found in the literature are carbon-based supports, such as carbon black, despite the corrosion problems [33]. To circumvent these issues, one of the most promising support materials that can be considered is graphene. Graphene is a 2D material composed only of a single layer of carbon atoms in a hexagonal pattern [34]. The exceptional strength and conductivity of these materials are ideal for energy and catalyst applications. However, most of the publications on catalysts use graphene oxide or reduced graphene oxide as support because the interactions between graphene and nanoparticles are weak [35]. Moreover, some synthesis of NPs over graphene uses solvothermal methods such as supercritical CO_2_ [36] that require expensive and sophisticated equipment. To easily improve the attachment of the NP over the surface of graphene, the use of surfactants can help in the formation of the composite and in the dispersion of the graphene to prevent agglomeration. Using surfactants is a common method to synthesize graphene [37]. Therefore, we decided to use tetrabutylammonium hexachloroplatinate (TBAH) as a simultaneous Pt precursor and tensioactive material to promote the attachment of the synthesized Pt_3_Cu NPs, and the dispersion of the graphene to improve the activity of the obtained catalyst.

Finally, the most important aspect of the present study is to bridge the obtained theoretical results and the experimental data here obtained. In particular, the main aim of this work is to use the knowledge from the previous theoretical studies to achieve a simple and reliable “one-pot” synthesis of Pt_3_Cu/graphene nanoplatelets (GNPp) and test them as efficient and versatile ORR catalysts.

This manuscript is organized as follows. In Section 2, the results of the theoretical study of the CPs of the MEP and the results of the physical and electrochemical characterization are presented along with a broad discussion of the obtained results. In Section 3, the computational details of the CP study and the synthetic and characterization methods are described. Finally, in Section 4, the conclusion of the present work is given with some perspectives for future work in this field of research.

## 2. Results and Discussion

### 2.1. MEP Bond Paths and Critical Points Search

The first attempts to describe the electronic structures and geometric properties of the molecules were provided by the valence theory of Lewis [38] and the valence shell electron pair repulsion (VSEPR) [39]. These theories are considered to achieve good results to predict the connectivity of the atoms in molecular systems and help to achieve a description of the metal and semimetal cluster bonding. An extension of these theories can be proposed using the topological analysis of scalar fields (such as MEP and density scalar fields) to calculate the bond CPs and find the corresponding bond path of molecules [40]. As already mentioned, good examples were published in the literature [28,30,31]. As discussed above, the MEP is a scalar field that can be experimentally observed, and therefore, the theoretically predicted results could serve as a guide for future synthetic approaches. Because of this, the CPs of the MEP of the (Pt_3_Cu)_n_, n = 1–11, clusters were computed here, and the obtained results will be discussed in this section.

The CP points obtained in the analysis can be characterized using their rank and the signature of the Hessian matrix (*H*), which can be listed as follows in terms of their signature:

(1) $$H_{\alpha \beta }={\left(\frac{\partial^{2}F\left(R\right)}{\partial R_{\alpha }\partial R_{\beta }}\right)}_{R=R_{CP}}$$

For the non-degenerated CPs, the rank of the critical points is always 3, which represents the number of non-zero eigenvalues of **H**. For these cases, there are four different types of CPs:

- (3, +3): local minimum (three positive eigenvalues)
- (3, +1): saddle point (two positive and one negative eigenvalues)
- (3, −1): saddle point (one positive and two negative eigenvalues)
- (3, −3): local maximum (three negative eigenvalues)

The above-given notations in round parenthesis mean rank and signature, i.e., (rank, signature), respectively. The (3, −3) points in the MEP appear at the nuclei of the atoms. Although they are not true CPs [41], their topological behavior is identical to a maximum. The CPs of type (3, −1) are very important in the topological analysis study since with them one can build a path between nuclear attractors. For our study, we only take into account the (3, −1) CPs that connect the nuclei. The paths that connect two maxima are called atomic interaction paths [42]. Due to the (3, −1) CPs and their atomic interaction paths connecting two nuclei, the (3, −1) CPs are called the bond CP and bond path, respectively.

As already mentioned, in Figure 1, the found lowest energy structures for the (Pt_3_Cu)_n_, n= 1–11 nanoclusters were presented. The structures used for this investigation were found performing a rigorous study based on molecular dynamics simulations in different electron spin multiplicities [20,21]. At the found optimized geometry the MEP CPs and bond paths were calculated.

**Figure 2:**
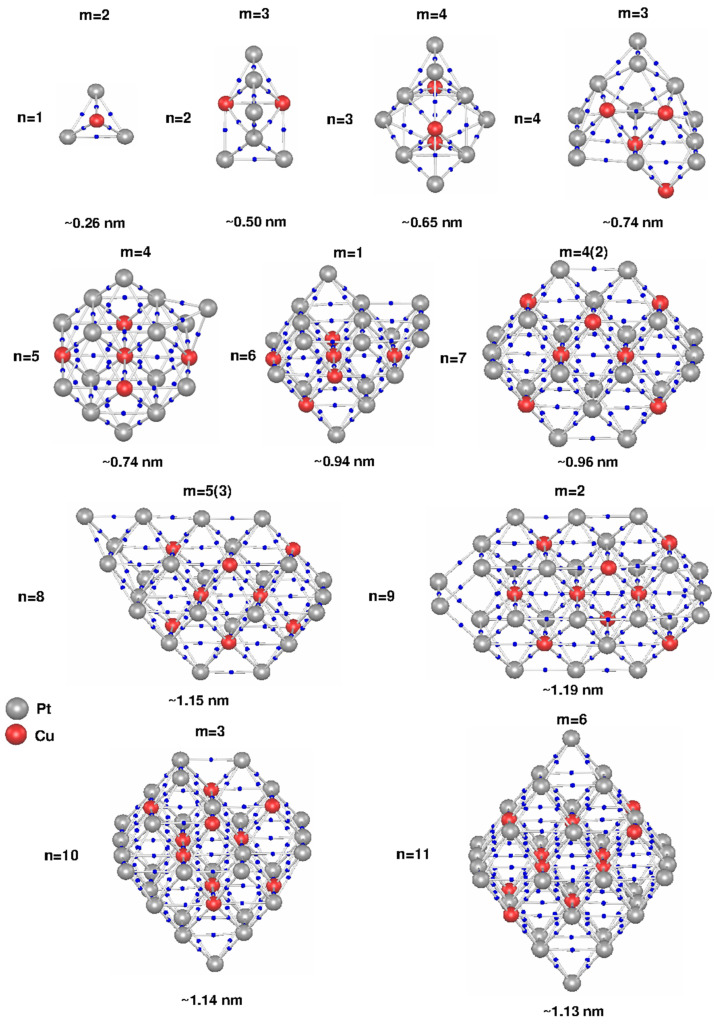
MEP molecular bond paths (gray lines) and MEP critical points (blue dots) of the (Pt_3_Cu)_n_, n = 1–11, clusters. The cluster electronic spin multiplicity, the number of initial Pt_3_Cu units with which each cluster size was initially built (n), and the approximate size of each cluster size are also given.

In Figure 2, the MEP CPs are depicted with blue dots and the MEP molecular bond paths are illustrated with gray lines, whereas the Cu and Pt atoms are represented with red and gray balls, respectively (see the legend of Figure 2 for more details).

It is important to note that the calculated molecular bond paths closely resemble the connectivity of the atoms depicted in the found most stable structures and presented in Figure 1, confirming the octahedral and twinned octahedral moiety formation in these clusters.

**Figure 3:**
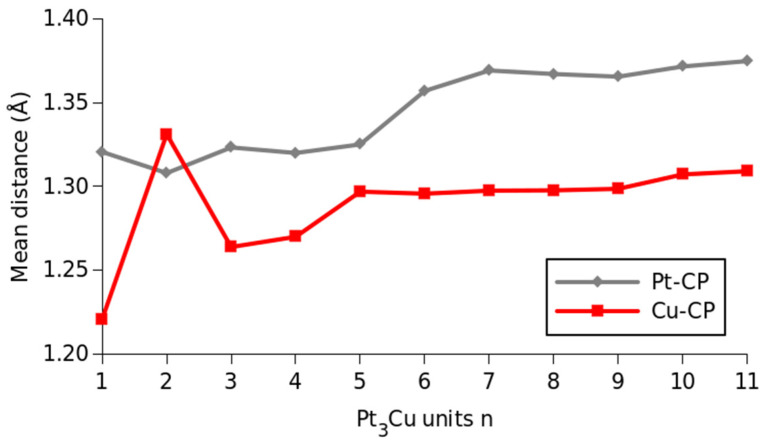
Mean distance of the Pt–CP and Cu–CP for each cluster size. The Pt–CP distance is plotted with a gray line, whereas the mean Cu–CP distance is illustrated with a red line. All values are given in Å.

To obtain an idea of the distribution of the CPs calculated from the MEP and of how far they are located from the Cu and Pt atoms in these cluster structures, the mean distances between the Cu and Pt atoms to the neighbor CPs ((Cu–CP) and (Pt–CP)) were calculated for each cluster size. The obtained results are plotted in Figure 3. As can be seen in Figure 3, the mean Pt–CP distance presents two different regions, one within the (Pt_3_Cu)_n_, n = 1–5, cluster size range, where the mean distance is around 1.32 Å. Then, a change in the mean distance is observed in the (Pt_3_Cu)_n_, n = 6–11, cluster range, where the mean distance increases to approximately 1.37 Å (see Figure 3). The behavior of the Cu–CP mean distance is different, showing an oscillating behavior at the first three cluster sizes. Then, the mean distance grows, obtaining an almost constant value of about 1.30 Å at the (Pt_3_Cu)_n_, n = 5–11, cluster range (see Figure 3).

The almost constant behavior of the mean Pt–CP and Cu–CP distances in the (Pt_3_Cu)_n_, n = 6–11 clusters, further support the existence of the octahedral moieties in these systems. However, this hypothesis does not explain the Pt–CP mean distance in the (Pt_3_Cu)_5_ cluster, which has an octahedral moiety but presents a different Pt–CP distance value concerning the one calculated for the bigger clusters. Probably, this difference is a result of the dangling atom at the face of the cluster that lowers the mean distance for the Pt atoms.

The Cu–CP constant mean distance observed in the n = 5–11 cluster size range can have also other explanations related to the “cross pattern” and “T pattern” formed by the Cu atoms that we previously observed for these clusters [20,21]. As can be observed in Figure 2, as the cluster size increases, the number of the CPs of the MEP grows considerably. To gain more insight into the bonding situation of these systems, in Table 1, the maximum coordination numbers (CN) for the Cu and Pt atoms for each cluster size are resumed.

As can be seen in Table 1, the general trend for the CN is to increase with the cluster sizes. Similar to the trend we just discussed for the calculated CPs mean distances, we note that the CNs of the clusters also characterized by the observed octahedral and twinned octahedral moieties, i.e., the (Pt_3_Cu)_n_ clusters, n = 5–11, tend to remain at a constant value.

Looking in more detail at the CNs trends, for the Cu atom the lowest maximum coordination number is 3 and it occurs for the (Pt_3_Cu)_1_ cluster. Then, it grows to 5 in the (Pt_3_Cu)_2_ and (Pt_3_Cu)_3_ cluster structures. Is important to note that up to this point, the CNs are the same for Pt and Cu atoms. Then, in the (Pt_3_Cu)_4_ cluster, the CN for the Cu atoms grows to a value of 6 (see Table 1). For the (Pt_3_Cu)_n_, n = 5–11 cluster size range, a constant value of 12 is then achieved for the CN of the Cu atom. This result could be explained considering that the central atoms of these clusters have a coordination number of 12, and all the central atoms of these structures are copper atoms. The initial hypothesis we made in our previous theoretical study was that Cu atoms form cross-type patterns to achieve the greatest number of Cu-neighboring atoms that occupy the central positions of the cluster [20,21]. Therefore, the here obtained results of the analysis of the CPs of the MEP confirm that the Cu atoms prefer to have the coordination number as high as possible, and this is the reason why the Cu atoms are arranged in peculiar patterns within the Pt_3_Cu cluster structures. Furthermore, it is also important to underline here that, in general, no Cu atoms are found at the vertex positions of these cluster structures, where the coordination number is the lowest.

For the Pt atom, as stated above, the CN of the first cluster is equal to 3, which is the same as for the CN of the Cu atom. Then, the CN value of platinum grows to 5 for the (Pt_3_Cu)_4_ cluster and then to 8 in the (Pt_3_Cu)_5_ cluster. Finally, the CN of the Pt atoms reaches a constant value of 9 for the (Pt_3_Cu)_n_, n = 6–11 cluster sizes. The Pt atoms occupy positions mostly at the faces, vertices, and apexes of the cluster structures, with coordination numbers varying between 3 and 9, which are lower than the ones already discussed for the central atomic positions that are occupied by Cu atoms in these systems.

Another interesting feature we observe in these clusters, considering the electronic spin density plots we obtained in our previous theoretical studies [20,21]. is related to the fact that the Pt atoms prefer lower coordination numbers and are characterized by high spin density, whereas the Cu atoms prefer higher coordination numbers but are characterized by low spin density.

The Pt–CP and Cu–CP mean distances, as well as the highest coordination number of the larger clusters, can be compared with the data from the literature [39]. The metallic radius, considering a coordination number of 12, for the Pt and the Cu atoms, are 137 and 128 pm, respectively [39]. In our study, the highest coordination number of 12 corresponds to mean Pt–CP and Cu–CP distances of 137 pm and 130 pm, respectively, which are in very good agreement with the metallic radius of the Pt and Cu atoms with this coordination number. The coordination number of 12 is also indicative of a closed-packaged cell system, such as a face-centered cubic (fcc) or Fm-3m space group, which, as we will discuss later, is the obtained space group for the Pt_3_Cu NPs synthesized here. Moreover, it is also important to note that this coordination number is the same as in the recently found orthocuproplatinum mineral, with the same system stoichiometry [43]. Until now, even with the information of the maximum coordination number was not possible to determine whether the clusters adopt the NP crystallographic symmetry of Fm-3m or the rhombohedral Cmmm symmetry observed in the orthocuproplatinum mineral. To be able to clarify this point, other experimental analyses are needed, as will be discussed in the next section.

### 2.2. Physical Characterization

#### 2.2.1. X-ray Diffraction Analysis (XRD)

**Figure 4:**
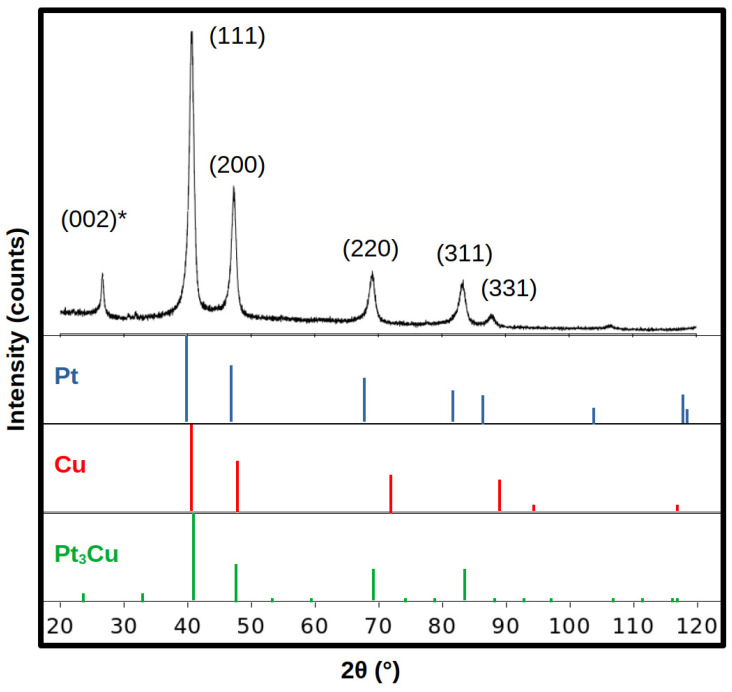
XRD pattern of the Pt_3_Cu/GNPp composite compared with the JCPDS of Pt (00-004-0802), Cu (00-004-0836), and Pt_3_Cu (04-017-6718). The crystallographic planes of each major peak are also described. The (002)* peak is assigned to graphitic materials.

Figure 4 presents the XRD pattern of the Pt_3_Cu/GNPp composite, along with the crystallographic plane that generates each major peak. The peak at 26.61° of 2θ is assigned to the graphitic materials (002) plane and is marked with an asterisk (*). The other major peaks were obtained at 40.63°, 47.30°, 69.07°, 83.23°, and 87.82° of 2θ for the (111), (200), (220), (311), and (331) planes, respectively.

Compared to the Pt and Cu diffraction patterns, the obtained Pt_3_Cu/GNPp have the same symmetry group and their major peaks are at a 20 angle intermediate from the Pt and Cu peaks. This is indicative of alloying between both species. The Pt_3_Cu of the JCPDS 04-017-6718 has a different spatial group than the here obtained material and the orthocuproplatinum mineral. There has been some debate about the true space group of the Pt_3_Cu alloy [43]; therefore, there is not a real guide to actually discover whether the space of the obtained NPs are the correct for this alloy.

The results of the Pt_3_Cu alloy XRD analysis are summarized in Table 2.

From Vegard’s law, it was found that the atomic fraction of the Pt and Cu are very close to the expected for a Pt_3_Cu alloy.

The XRD pattern shows that the GNPp presents a small degree of restacking, thus obtaining graphite, as demonstrated by the (002) peak at the 26.61° of 2θ. This is normal, as the GNPp tends to restack over time, and considering the size of the peak and the intensity, it can be concluded that most of the GNPp is well separated and stabilized.

The space group of the alloy is the same as the constituent metals, and from cell parameters, Vegard’s law is able to estimate the concentration of the Pt material. It was found that the Pt and Cu atomic fraction is similar to that of the expected alloy. The crystallite size obtained by the Scherrer’s Law is 8.02 mm, which in the NPs are roughly equivalent to the expected size of the NPs.

Comparing the results of the XRD analysis to the theoretical results is found that the atomic radius of the alloy of 0.1358 nm is in between the mean distance calculated for the Pt–CP (0.137 nm) and Cu–CP (0.130 nm), also further validating the theoretical results of the (Pt_3_Cu)_n_, n = 1–11, clusters as a reliable source of information for this alloy. Moreover, the higher coordination number obtained in the theoretical results of 12 matches with an fcc structure, which is the Fm-3m symmetry obtained in the XRD analysis of this work.

#### 2.2.2. BET Surface Area Measurement

**Figure 5:**
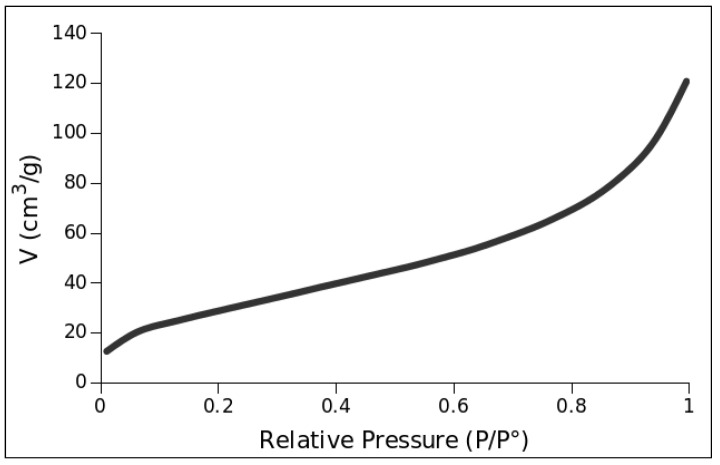
Isotherm of the Pt_3_Cu/GNPp material.

The BET surface area technique was employed to measure the surface area of the prepared Pt3Cu/GNPp material. Figure 5 shows the obtained isotherm for the composite.

Figure 5 shows a type II isotherm which is characteristic of macroporous materials, i.e., a wide range of porous sizes. This result is expected from graphitic materials. The obtained surface area is 110.03 m^2^/g for a multipoint determination. The pore total volume is 0.187 cm^3^/g. This area can be compared to the reagent specification for the GNPp of minimum 500 m^2^/g. The results show that the synthesis and the NP deposition over GNPp diminishes fivefold in the total surface area, as observed by the XRD peak at 26.61° of 2θ, which shows some degree of restacking and agglomeration.

#### 2.2.3. Scanning Electron Microscopy and Energy Dispersive Spectroscopy

**Figure 6:**
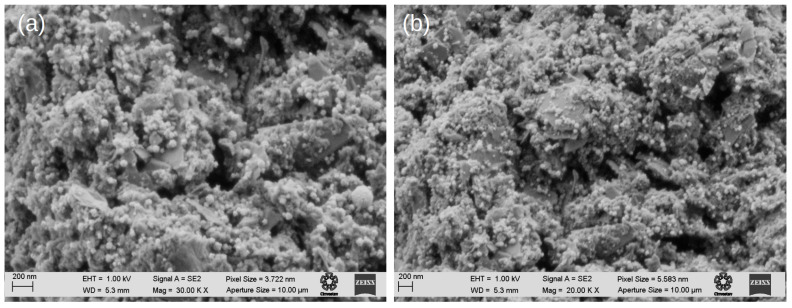
(**a**,**b**) SEM images of the Pt_3_Cu/GNPp composite. The NPs appear as white dots over the surface of the graphene.

The catalyst obtained is characterized using scanning electron microscopy (SEM). In Figure 6a,b, it can be observed that the NPs appear as white dots uniformly dispersed over the graphene. Additionally, from the images it can be seen that the borders of the GNPp are well-defined, furthermore supporting the information of the XRD analysis.

The graphene nanoplatelet (GNPp) edges appear intact, suggesting that the graphitic nature of the material is conserved, and most of the NPs are shown to attach to the GNPp surface.

To find whether the correct material was obtained, an EDS analysis was performed. Table 3 shows the obtained results for the unnormalized and normalized weight percentage (wt%) and the normalized atomic percentage (at%).

The table shows Pt, Cu, and C as the major components of the synthesized catalyst. Additionally, the material has a small oxidation percentage from the GNPp. A small contamination of K and Al was also observed.

From the at%, the ratio of Pt to Cu is 2.71, which is near that expected from a Pt_3_Cu alloy. Moreover, by calculating the percentage of the Pt and Cu wt%, considering the sum of Pt, Cu, and C wt%, a 47.63% metal loading is obtained, which is expected from the originally planned 50% from our synthesis. The obtained Al and K contamination of the EDS analysis is without importance due to the semi-quantitative nature of the EDS analysis. An important absence in the EDS analysis is the nitrogen that could anchor the NPs to the surface of GNPp if the anchoring mechanism is to form non-covalent functionalized graphene. Interestingly, an excess of tetrabutylammonium bromide (TBAB) is required to disperse and even reduce the metals over the surface of the GNPp. It has been found that at lower temperatures (<200 °C), the formation of NPs simply does not occur. The TBAH has a higher reduction temperature than the Cu(acac)_2_, which are the precursors of the present Pt_3_Cu NPs. The TBAB does not affect the reduction temperature of the TBAH alone; however, in the presence of GNPp, the reaction proceeds at a lower temperature if enough TBAB is added. Currently, the true function of the TBAB that permits the reaction at a lower temperature is not known, as is unknown whether the TBAB has an anchoring function. The EDS analysis and the CV profile, discussed below, do not support the idea of non-covalent anchoring and the functionalization of GNPp. A benchmark analysis that can help to uncover whether the interaction is strong enough to functionalize the GNPp is using the TBAB as a stabilizing agent for the graphene synthesis from graphite using the liquid phase exfoliation. The tetrabutylammonium cation has been used successfully with electrochemical exfoliation methods [44]. Therefore, one of the hypotheses is that the TBAB stabilizes the GNPp in the oleylamine media and reduces the surface tension of the GNPp, augmenting their reactivity to form and anchor the NPs over the surface of the GNPp. Further studies are required to find this mechanism.

#### 2.2.4. Transmission Electron Microscopy

**Figure 7:**
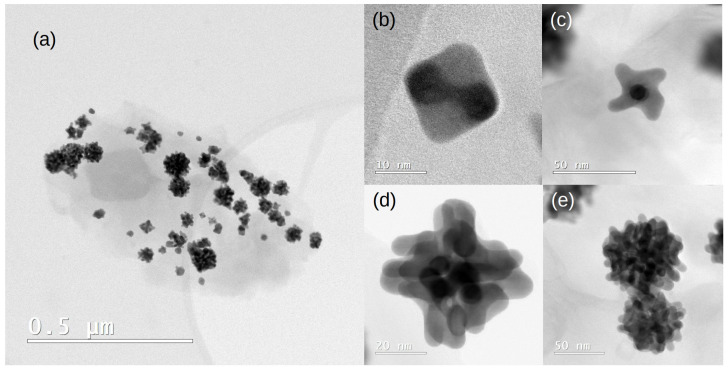
TEM images of the obtained material (**a**) Pt_3_Cu NP over the GNPp. (**b**) Below approximately 14 nm, the octahedron is the preferred geometry. (**c**,**d**) As the NP starts to grow, dendrons are formed over the surface. (**e**) Final form of the NP as nanoflowers.

To find the geometry of the NPs, TEM image analysis was performed. Figure 7a shows a Pt_3_Cu/GNPp, in which the dispersion of the NPs over the GNPp is non-homogeneous. The images demonstrate a distribution of different sizes and shapes of the NPs. Furthermore, inspecting the NPs, we found that the smaller sizes have octahedral moieties below 14 nm, and the bigger sizes around 50 nm NPs have a dendritic “nanoflower” shape. Between these sizes, intermediate geometries were found.

TEM images show different geometries at different NPs sizes. From these images, a growth mechanism can be speculated: the NP’s initial geometry is octahedral, as seen in Figure 7b, and then starts to grow initially at the apexes, forming dendrons and displaying the geometry of Figure 7c. Then, dendrons start to grow around all the NP surfaces, as observed in Figure 7d. Finally, above 50 nm, the NPs obtain a final nanoflower shape. Similar nanoflower geometries were synthesized and reported in a different synthetic media [15]. However, a similar approach, using a one-pot method to simultaneously synthesize and support the Pt_3_Cu NPs over GNPps was performed, using different reagents, no tensioactive and using water as a solvent, controlling the pH of the media. A simple explanation is that the GNPps promote the dendritic mechanism and are somewhat slowed by the TBAB of our synthesis to find the octahedral moieties, until the NP becomes too big and the dendritic growth appears. This hypothesis should be validated in future studies.

It is of paramount importance to note that the octahedral structures were predicted by the theoretical studies, and a growth mechanism involving octahedral twinning moieties was also predicted [20,21]. However, a transition was observed, and the twinning mechanism can also be seen as a one-directional dendritic growth mechanism. The reason for seeing twinning structures in the theoretical studies and not finding them in the synthesized material is probably due to the limitation of simulating small clusters compared to the actual NPs (approximately 1.13 nm vs. 14 nm in size). Therefore, an essential perspective of this study is simulating bigger clusters to try to find the dendritic growth mechanism.

**Figure 8:**
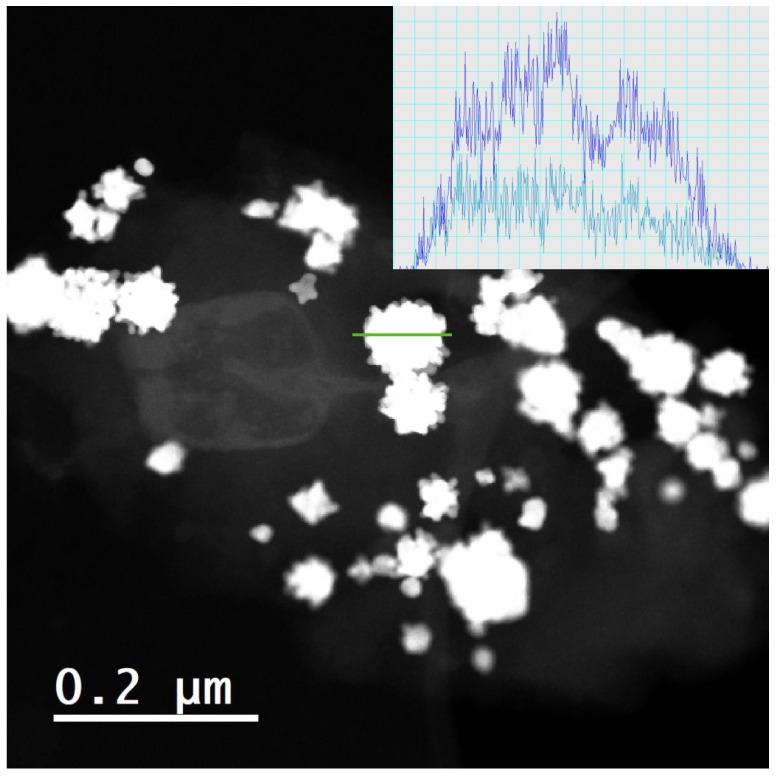
HRTEM-EDS profile of the obtained material. The green line marks the area where the EDS profile analysis has been performed. Insert: Pt (purple) and Cu (blue) quantities found in the area.

Figure 8 presents the HRTEM-EDS profile of the obtained nanoparticles. For better clarity, the oxygen and carbon signals have been subtracted. It is found that the alloying is evenly distributed in the nanoparticle. The approximate atomic quantities of the material are near to the ones expected for the Pt_3_Cu alloy.

### 2.3. Electrochemical Characterization

**Figure 9:**
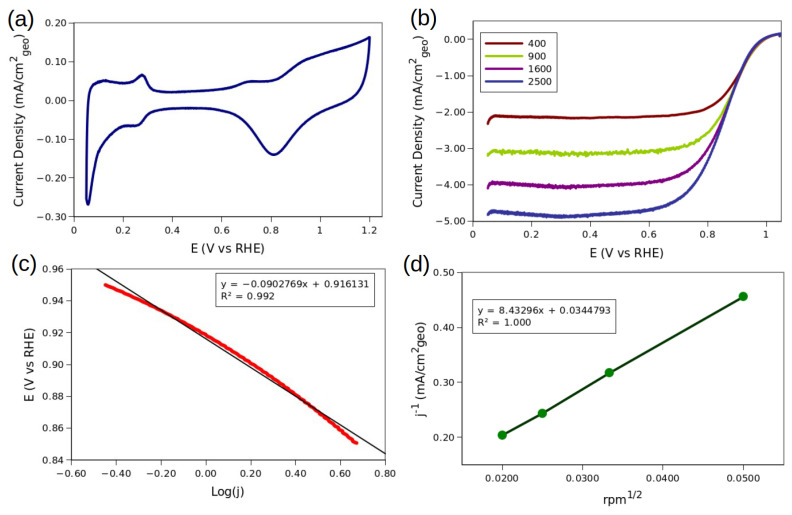
(**a**) Cyclic voltammetry of Pt_3_Cu/GNPp. (**b**) Hydrodynamic voltammetry of Pt_3_Cu/GNPp in saturated O_2_ electrolyte, from which the anodic sweep is only presented. (**c**) Tafel curve of the ORR with their slope. (**d**) Koutecky–Levich analysis of the ORR with their slope.

Figure 9 shows the main results of the electrochemical characterization. Cyclic voltammetry experiments were used to obtain a profile of the material that is similar to the reported for a Pt-based alloy. Is important to note the characteristic signal at 0.28 V vs. reversible hydrogen electrode (RHE), which is assigned to the adsorption of the H species over <111> planes. Additionally, a small oxidation signal is found at 0.7 V vs. RHE, and it corresponds to the graphene [45].

Figure 9b presents the hydrodynamic voltammetry curves of the catalyst into the O_2_ saturated solution at 400, 900, 1600, and 2500 revolutions per minute (rpm). An even distribution of the curves and a flat section below 0.6 V vs. RHE can be observed, indicating that the diffusion-limited current is reached. From these curves, a Tafel and Koutecky–Levich analysis was performed, and the resultant plots are presented in Figure 9c,d, respectively. 

The CV profile obtained In Figure 9a has the characteristics of a Pt-based material profile. The signals at 0.28 V vs. RHE are assigned to the <111> plane H adsorption [46]. These planes are common for octahedra-type structures, supporting the results obtained in the TEM analysis. The other interesting aspect of the voltammogram is the wide peak found around 0.7 V vs. RHE. A similar peak has been found experimentally in the reduced graphene oxide. This is probably owed to the oxidized GNPps that are near the surface of the electrode. Another possible explanation is that the GNPps transfer oxides or even carbonyl groups to the Pt. This possibility is supported because of the potential of this artifact is near to the CO electro-oxidation over pure Pt [47]. The CV profile is substantially different from other Pt/graphene composites, showing a sharper figure compared to the materials reported in the literature [48]. The narrow capacitive zone of around 0.4 to 0.6 V vs. RHE is like the Pt over carbon materials, such as the carbon Vulcan.

The hydrodynamic voltammetry of the material shows an even distribution of the curves and a flat, mass transfer limiting current below 0.6 V/RHE. To the best of our knowledge, this is the first GNPp catalyst that displaysx this behavior towards the ORR reaction in acidic media. The electrochemical results are like carbon-supported Pt-based catalysts. Comparing these results with some benchmark results, the activity is lower than some Pt/C materials with similar metal loadings [49]. However, the results are very good from a material supported in GNPps, as it is difficult to anchor the NPs over the surface of graphene. The material has lower mass activity however, and the ECSA or surface activity is comparable to the Pt/C materials, probably due to the smaller particle size of the Pt/C materials.

The final results of the activity regarding the ORR are provided in Table 4 and compared with Pt/C 20% Etek. 

Is important to note that the Pt/C outperforms the graphene material in every value, which is due to the nature of the carbon black. Carbon black has many defects and oxygen-based functional groups that effectively interact and anchor the NPs. Additionally, the Pt/C have a smaller NP size, which explains the much higher ECSA than the obtained material. Is also important to note that the BET values are much higher than the ECSA values, which demonstrates that most of the area are the GNPp, which are not active in regard to the CO-stripping testing. 

The inverse of the Koutecky–Levich slope shows a value very near to the B = 0.1241 mA/cm^2^, which is theoretically obtained at sea level. However, all the present experiments were conducted in Mexico City, at 2200 m over sea level, where the oxygen concentration in electrolytes is lower than at sea level. Therefore, the B obtained is close enough to the theoretical value that almost all the O_2_ that the Pt_3_Cu/GNPp reduces is with the four-electron pathway:

(2) $$O_{2}+4H^{+}+4e^{-}\to 2H_{2}O$$

### 2.4. Electrochemical Stability Test

**Figure 10:**
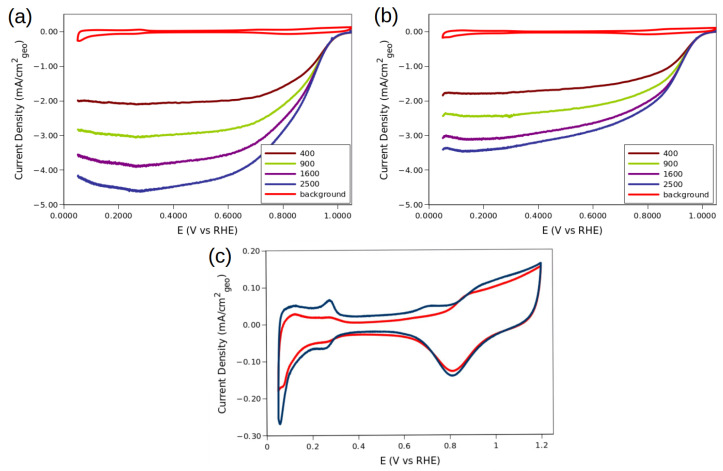
Hydrodynamic voltammetry of Pt_3_Cu/GNPp in saturated O_2_ electrolytes (**a**) before and (**b**) after 10,000 cycles. The anodic sweep is presented, only (**c**) CV profiles of the catalyst before (blue) and after the 10,000 cycles (red).

The principal attractive of using GNPp as support for the metal NPs is their stability. The material was tested for over 10,000 cycles to uncover whether the activity was diminished. Figure 10a,b presents the hydrodynamic voltammetry curves for the Pt_3_Cu/GNPp. The CV profile of the composite is also added in red, to compare the techniques. It can be observed that the curves reach a lower mass-transfer limiting current after testing. However, most of the activity at lower overpotentials, i.e., above 0.90 V, are still retained. The 2500 rpm curve is the most affected as their limiting current is similar to the 1600 rpm curves. Due to the nature of the mass transfer current, the hypothesis of this behavior is that the composite becomes a more dense agglomerate, hindering the ability of the oxygen to permeate into the inner parts of the ink layers. 

Also, the NPs probably agglomerates instead of degrading, which explains the similar activities at 0.90 V vs. the RHE of the “fresh” and “old” materials. An observation that supports this hypothesis is the lack of the signals at 0.28 vs. the RHE in the CV profiles of the materials (see Figure 10c) corresponding to the <111> faces in the CV profile of the material after 10,000 cycles. Another important reason why the limiting current becomes smaller is because of the very inhomogeneous sizes of the obtained NPs, with the smaller more likely to corrode and fail, in comparison to the bigger size NPs. This aspect, with the different geometries, could be the reason why this behavior is found, as some geometries would be more stable than others [20,21].

The final results of the stability test are given in Table 5. As discussed earlier, most of the mass activity is retained, with a modest ca. 10% loss in activity. The loss of about 25% of ECSA is also a result of the agglomeration discussed above. These ECSA loss is the reason why the specific activity of the Pt_3_Cu/GNPp is higher after 10,000 cycles.

The Tafel and the Koutecky–Levich slope yields similar results in both tests, from which it can be concluded that the 4e^−^ mechanism still is dominant after the stability test.

## 3. Materials and Methods

### 3.1. Computational Details

All the calculations were performed using a linear combination of Gaussian type orbitals in the Kohn–Sham auxiliary density functional theory, as coded in the deMon2k software [50,51]. The Coulomb integrals were calculated using the variational fitting proposed by Dunlap, Conolly, and Sabin [52,53].

For the correlation functional, the original Perdew–Burke–Ernzehof functional was employed [54], and for the exchange functional, the revised Perdew–Burke–Ernzehof functional by Yang and Parr was used (RPBE) [55]. The optimized structures of the (Pt_3_Cu)_n_, n = 1–11, were taken from Refs. [20,21] to calculate the MEP molecular graphs and to obtain the CPs from the MEP of these systems. For the Cu atoms, a triple zeta basis set optimized for GGA functionals, such as PBE and RPBE (TZVP-GGA) [56], was employed, whereas for the Pt atom, the original quasi-relativistic effective core potential basis set was replaced with an all-electron triple zeta basis set with Douglas–Kroll–Hess scalar relativistic effects [57]. The GEN-A2* auxiliary function set was employed.

The search for the CPs and the calculation of the bond paths were performed using the implemented algorithm in deMon2k [58]. This algorithm is used to find CPs of all kinds of scalar fields and is described in the literature [30].

The plots of the CPs obtained from the MEP were performed with the VuChem software [59]. The mean Cu–CP and Pt–CP distances were obtained using an Octave program made by the authors [60].

### 3.2. Synthesis Methods

- TBAH synthesis: The method used has been reported in the literature with minimal modifications [61]. Equimolar quantities of K_2_PtCl_6_ (Sigma-Aldrich, St. Louis, MO, USA, 98%) and TBAB (STREM Chemicals, Newburyport, MA, USA, 99%) were weighed and dissolved in water and chloroform, respectively. The TBAB solution was poured into the K_2_PtCl_6_ and was left under vigorous agitation for 5–6 h or until the aqueous phase became transparent. The organic phase was separated and distilled, from which a further recrystallization was performed in acetone.
- Pt_3_Cu/GNPp composite synthesis: In a three-way flask, 0.0100 g of GNPps (STREM Chemicals), 0.0413 g of the prepared TBAH (0.0465 mmol), 0.0041 mg of copper acetylacetonate (Sigma-Aldrich, ≥99.9%, 0.0155 mmol), and 0.0250 mg of TBAB were added to and dissolved in 10 mL of oleylamine (SAFC, Darmstadt, Germany, ≥98%). The reaction mixture was heated at 120 °C for 30 min into N_2_ (Infra, ultrapure) inert atmosphere to extract all the water and oxygen present in the mixture, and then the mixture was heated to 250 °C for 15 min. The N_2_ atmosphere was conserved in all the experiment until the mixture was cooled to room temperature. After the heating time, the mixture was left to cool and washed using a blend of ethanol:hexane. The first washing began with pure ethanol, and then the mixture was centrifugated at 12k rpm for 10 min to precipitate the composite. The washing process was then repeated, augmenting the hexane concentration until the composite was dispersed and precipitated in pure hexane. The composite was left in an oven to dry at 80 °C overnight. A black powder similar to the GNPps was finally obtained.
- Electrochemical Ink Formulation: The obtained Pt_3_Cu/GNPp composite was extracted of the oven and left to cool. A total of 3.6 mg of the material was dispersed in a mixture of ultrapure miliQ water (18.2 MΩ) in acetone (Sigma-Aldrich) at 20% in volume. This concentration was obtained from an experiment of graphene liquid phase exfoliation as the best mixture to disperse graphene [62], and it was also able to disperse graphene composites. A total of 5 μL of Nafion solution (Sigma-Aldrich, 5 wt%) was added to the ink formulation. The ink was sonicated for 1 h in a bath with ice to prevent the heating of the ink.
- Electrochemical Technique: A total of 20 μL of the ink explained above was deposited into a glassy carbon electrode (7 mm, 0.3848 cm^2^) and left to dry. No rotation was required to dry the electrode. The ink-type electrode was tested in a three-electrode electrochemical cell. A Pt foil was used as the counter electrode, and a Pt wire was used to form an H_2_ bubble electrolytically, which served as the RHE. All experiments were performed in a solution of 0.1 M HClO_4_ (Sigma-Aldrich, 70 wt%) in miliQ water, bubbling with N_2_ in order to remove all oxygen from the solution in the profile and CO-stripping experiments and O_2_ (Infra, ultrapure) in the ORR hydrodynamic voltammetry. All experiments were conducted with a Metrohm PGSTAT 302N potenciostat.
- Activation CV: The material was electrochemically cleaned using cyclic voltammetry at 100 mV/s in a window of 0.05 V–1.2 V vs. RHE for 250 cycles until the CV was stable.
- Profile CV: The profile was obtained at 20 mV/s in a window of 0.05 V–1.2 V vs. RHE, after two cycles. The last cycle was selected and presented.
- CO stripping: The solution was bubbled with CO gas for 300 s with the electrode polarized at 0.1 V vs. RHE to promote the adhesion of the CO over the surface of the composite. This was followed by a vigorous bubbling of N_2_ for 300 s to remove all the CO that did not react with the solution. After the time passed, a CV experiment was performed at 20 mV/s in a window of 0.05 V–1.2 V vs. RHE, after two cycles. The last cycle was rested from the first cycle, and the resultant peak was integrated and converted into ECSA using a factor of 420 µC/cm^2^.
- Hydrodynamic ORR curves: The solution was saturated with vigorous O_2_ bubbling for 25 min. Then, the glassy carbon electrode was rotated using an electric motor at speeds of 400, 900, 1600, and 2500 rpm. A CV experiment was performed for each rotation at 20 mV/s in a window of 1.03 V–0.05 V vs. RHE after two cycles. The last anodic sweep was selected from all rotations. The Tafel slope was obtained from the 1600 rpm anodic sweep cycle.
- Stability testing: To assess the stability of the catalyst with respect to a similar behavior of a real fuel cell, 10,000 cycles of CV of 0.7–1.0 V vs. RHE were performed. The complete set of experiments described above (activation CV, profile CV, CO stripping, and hydrodynamic ORR curves) were performed on the catalyst before and after the test.

## 4. Conclusions

This study of the critical points obtained from the MEP demonstrates the growing pattern that leads to two (apparently) different geometries (octahedra and twinned octahedra), that are characterized by similar coordination numbers and similar mean X–CP (X = Cu, Pt) distances. These structures are influenced by the preference of the Cu atoms to have greater coordination numbers and by the Pt atoms to have a higher electronic spin density. The calculated metallic radius is very similar to the metallic radius reported in the literature for each atom and is similar to the atomic radius obtained in the XRD analysis. Additionally, the higher coordination number matches the crystallographic symmetry point group Fm-3m (i.e., fcc) obtained in the XRD experiment.

Using the previously obtained knowledge, the Pt_3_Cu/GNPp catalyst is synthesized using a simple one-pot reaction to simultaneously form the NPs supported over GNPp.

The physical characterization of the synthesized Pt_3_Cu alloy nanoparticles over the surface of GNPp shows the theoretically predicted octahedral geometries below 14 nm size. NPs that are bigger than 14 nm start to grow dendritically. Furthermore, the twinned octahedra structures can be seen as one-way dendritic growing, which supports the nanoflower formation obtained in the NPs of around 50 nm. An important direction for theoretical studies is to increase the size of the simulated clusters to uncover whether the twinned octahedral moieties match the dendritic growing pattern. However, this is outside of the scope of this paper.

EDS analysis demonstrates that the obtained material is near the Pt_3_Cu/GNPp of 50% in mass of GNPp and shows that the tetrabutylammonium cation does not anchor the NP with non-covalent functionalization. A future perspective in this field would be to understand the details of the mechanism of the anchoring of the NPs to the GNPp to further improve the catalytic activity and the use of graphene as support for the ORR.

The electrochemical measurements show that the material has improved activity over the Pt/graphene composites, presenting a behavior typical of the Pt/C materials, such as flat mass transport limiting current and low capacitive area. The activity of the material is comparable to the Pt/C materials of similar metal loading and has very good stability towards 10,000 cycles of ORR. The Tafel and the Koutecky–Levich analysis shows that the material is active towards ORR and most of the oxygen is reduced via a four-electron pathway mechanism ($O_{2}+4H^{+}+4e^{-}\to 2H_{2}O$).

Herewith, we presented a bridge between the results obtained from our theoretical studies with experimental and synthetic results. This link permitted us to achieve an effective, yet simple, synthetic approach to obtain a good catalyst towards the ORR. Therefore, we can conclude that the performed ab initio theoretical studies are a reliable source of information for the successful synthesis of the Pt_3_Cu alloy system.

## Figures and Tables

**Table 1:** Maximum coordination number obtained for the Cu and Pt atoms with the MEP for the (Pt_3_Cu)_n_, n = 1–11 clusters.

# of Pt_3_Cu Units (n)	1	2	3	4	5	6	7	8	9	10	11
**CN MEP Cu**	3	5	5	6	12	12	12	12	12	12	12
**CN MEP Pt**	3	5	5	5	8	9	9	9	9	9	9

**Table 2:** XRD results for the Pt_3_Cu alloy.

Symmetry	Cell Parameter a	Atomic Radius	Vegard Pt f	Vegard Cu f	Crystallite Size
Fm-3m	0.3843 nm	0.1358 nm	0.7427	0.2572	8.02 nm

**Table 3:** EDS composition of the sample.

Element	Series	Unnormalized C (wt%)	Normalized C (wt%)	Normalized C (at%)	Error (1σ, wt%)
Oxygen	K-series	1.91	2.23	2.99	0.41
Copper	K-series	4.31	5.02	1.69	0.17
Platinum	L-series	35.76	41.64	4.58	1.11
Potassium	K-series	0.27	0.31	0.17	0.04
Aluminum	K-series	0.12	0.14	0.11	0.03
Carbon	K-series	43.52	50.67	90.46	5.25

**Table 4:** Typical results for the Pt_3_Cu/GNPp @ 0.9 V vs. RHE, compared to commercial Pt/C Etek 20%.

Material	Mass Activity (mA/mg_Pt_)	ECSA (m^2^/g_Pt_)	Specific Activity (μA/cm^2^_Pt_)	Tafel Slope (mV/dec)	KL B (mA/cm^2^)
Pt_3_Cu/GNPp	53.1	22.13	194.9	90.2	0. 1186
Pt/C Etek 20%	222	68.6	324	68.9	0.1217

**Table 5:** Stability test results of the Pt_3_Cu/GNPp at 0.9 V vs. RHE after 10,000 cycles.

Material	Mass Activity (mA/mg_Pt_)	ECSA (m^2^/g_Pt_)	Specific Activity (μA/cm^2^_Pt_)	Tafel Slope (mV/dec)	KL B (mA/cm^2^)
Fresh	53.1	22.13	243.9	90.2	0.1186
After 10,000 cycles	48.1	17.06	285.5	94.2	0.1101

## Data Availability

All data are available upon request to the authors.

## Sample Availability

Samples of the composite are available from the authors.
